# Structure of Csd3 from *Helicobacter pylori*, a cell shape-determining metallopeptidase

**DOI:** 10.1107/S1399004715000152

**Published:** 2015-02-26

**Authors:** Doo Ri An, Hyoun Sook Kim, Jieun Kim, Ha Na Im, Hye Jin Yoon, Ji Young Yoon, Jun Young Jang, Dusan Hesek, Mijoon Lee, Shahriar Mobashery, Soon-Jong Kim, Byung Il Lee, Se Won Suh

**Affiliations:** a Department of Biophysics and Chemical Biology, College of Natural Sciences, Seoul National University, Seoul 151-742, Republic of Korea; b Department of Chemistry, College of Natural Sciences, Seoul National University, Seoul 151-742, Republic of Korea; c Research Institute of Pharmaceutical Sciences, College of Pharmacy, Seoul National University, Seoul 151 742, Republic of Korea; d Department of Chemistry and Biochemistry, University of Notre Dame, Notre Dame, IN 46556, USA; e Department of Chemistry, Mokpo National University, Chonnam 534-729, Republic of Korea; f Biomolecular Function Research Branch, Division of Convergence Technology, Research Institute, National Cancer Center, Gyeonggi 410-769, Republic of Korea

**Keywords:** *Helicobacter pylori*, *csd3*, HP0506, peptidoglycan hydrolase, cell-shape determinant, d,d-endopeptidase, d,d-carboxypeptidase, M23B family metallopeptidase, LytM

## Abstract

*Helicobacter pylori* is associated with various gastrointestinal diseases such as gastritis, ulcers and gastric cancer. Its colonization of the human gastric mucosa requires high motility, which depends on its helical cell shape. Seven cell shape-determining genes (*csd1*, *csd2*, *csd3*/*hdpA*, *ccmA*, *csd4*, *csd5* and *csd6*) have been identified in *H. pylori*. Their proteins play key roles in determining the cell shape through modifications of the cell-wall peptidoglycan by the alteration of cross-linking or by the trimming of peptidoglycan muropeptides. Among them, Csd3 (also known as HdpA) is a bifunctional enzyme. Its d,d-endopeptidase activity cleaves the d-Ala^4^-*m*DAP^3^ peptide bond between cross-linked muramyl tetrapeptides and pentapeptides. It is also a d,d-carboxypeptidase that cleaves off the terminal d-Ala^5^ from the muramyl pentapeptide. Here, the crystal structure of this protein has been determined, revealing the organization of its three domains in a latent and inactive state. The N-terminal domain 1 and the core of domain 2 share the same fold despite a very low level of sequence identity, and their surface-charge distributions are different. The C-terminal LytM domain contains the catalytic site with a Zn^2+^ ion, like the similar domains of other M23 metallopeptidases. Domain 1 occludes the active site of the LytM domain. The core of domain 2 is held against the LytM domain by the C-terminal tail region that protrudes from the LytM domain.

## Abstract 2

*H. pylori* Csd3 (HP0506), together with other peptidoglycan hydrolases, plays an important role in determining cell shape. Its crystal structure in the latent state is reported.

## 1. Introduction   

*Helicobacter pylori* is a spiral-shaped Gram-negative bacterium that colonizes the human stomach. It colonizes approximately half of the world’s population and its infection of the gastric mucosa has been associated with various diseases of the upper gastrointestinal tract, such as chronic gastritis, peptic ulcer, mucosa-associated lymphoid tissue (MALT) lymphoma and gastric adenocarcinoma (Roesler *et al.*, 2014 ▶; Kusters *et al.*, 2006 ▶). *H. pylori* has been classified as a group I carcinogen by The International Agency for Research on Cancer and is regarded as a primary factor in the development of gastric cancer (International Agency for Research on Cancer, 1994 ▶). In recent years, *H. pylori* infection has also been implicated in some extra-digestive diseases (Roubaud Baudron *et al.*, 2013 ▶). Relatively effective treatment regimens are available for *H. pylori* infection that typically consist of a proton-pump inhibitor such as omeprazole and the antibiotics clarithro­mycin and amoxicillin (or metronidazole). However, increasing antibiotic resistance requires new therapies and the discovery of new antibiotics (Malfertheiner *et al.*, 2012 ▶).

High motility of *H. pylori* is important for its colonization of the human stomach and its survival in the gastric mucosa (Ottemann & Lowenthal, 2002 ▶; Schreiber *et al.*, 2004 ▶; Lertsethtakarn *et al.*, 2011 ▶). The spiral or helical cell shape of *H. pylori* is believed to facilitate efficient colonization of the viscous epithelial mucus layer *via* a corkscrewing mechanism (Berg & Turner, 1979 ▶; Hazell *et al.*, 1986 ▶; Worku *et al.*, 1999 ▶). *H. pylori* mutants with altered cell shapes exhibit attenuated colonization (Bonis *et al.*, 2010 ▶; Sycuro *et al.*, 2010 ▶, 2012 ▶; Frirdich *et al.*, 2012 ▶; Wyckoff *et al.*, 2012 ▶). The peptidoglycan layer of the bacterial cell wall plays a role not only in withstanding the turgor pressure but also in maintaining the cell shape (Scheffers & Pinho, 2005 ▶; Vollmer & Bertsche, 2008 ▶). An essential component of bacterial peptidoglycan is a linear polysaccharide chain consisting of alternating β-1,4-linked *N*-acetylglucosamine (NAG) and *N*-acetylmuramic acid (NAM) disaccharides, with a pentapeptide linked to NAM (Vollmer, Blanot *et al.*, 2008 ▶). In helical-shaped *H. pylori*, the pentapeptide sequence is l-Ala^1^-γ-d-Glu^2^-*m*DAP^3^-d-Ala^4^-d-Ala^5^, where *m*DAP refers to *meso*-2,6-diaminopimelate and the neighbouring peptides are cross-linked exclusively by the 4→3 linkage between the main chain of d-Ala^4^ from one strand and the side chain of *m*DAP^3^ from another strand (Costa *et al.*, 1999 ▶) to form a mesh-like structure (Meroueh *et al.*, 2006 ▶). In many bacteria, the peptidoglycan layer is re­modelled by a number of cell-wall hydrolases as well as synthetases for peptidoglycan maturation, regulation of cell-wall growth, cell division, peptidoglycan turnover and recycling, cell lysis and the release of peptidoglycan fragments for host–pathogen interactions (Vollmer, Joris *et al.*, 2008 ▶).

At least seven *H. pylori* genes have been identified to be required for determining the helical cell shape: *csd1*, *csd2*, *csd3*/*hdpA*, *ccmA*, *csd4*, *csd5* and *csd6* (Sycuro *et al.*, 2010 ▶, 2012 ▶, 2013 ▶; Bonis *et al.*, 2010 ▶). They play important roles in determining the helical cell shape of *H. pylori* by the relaxation of peptidoglycan cross-linking or by the trimming of pentapeptides to shorter peptides in peptidoglycan. Among them, the Csd3/HdpA protein as well as Csd1 and Csd2 belong to the MEROPS M23B metallopeptidase family (Sycuro *et al.*, 2010 ▶; Bonis *et al.*, 2010 ▶). Deletion of the *csd1*, *csd2* and *csd3* genes reduced the d,d-endopeptidase (d,d-EPase) activity, which cleaves the d-Ala^4^-*m*DAP^3^ peptide bond in cross-linked dimers of muramyl tetrapeptides and pentapeptides (muramyl-l-Ala^1^-γ-d-Glu^2^-*m*DAP^3^-d-Ala^4^ and muramyl-l-Ala^1^-γ-d-Glu^2^-*m*DAP^3^-d-Ala^4^-d-Ala^5^, respectively; Bonis *et al.*, 2010 ▶; Sycuro *et al.*, 2010 ▶).

Interestingly, the muropeptide composition of the deletion mutant of the *csd3* gene (Δ*csd3*) indicated that Csd3 has an additional d,d-carboxypeptidase (d,d-CPase) activity that cleaves the d-Ala^4^-d-Ala^5^ bond of the muramyl pentapeptide to produce the muramyl tetrapeptide and d-Ala (Bonis *et al.*, 2010 ▶; Sycuro *et al.*, 2010 ▶). In accordance with this observation, Δ*csd3* showed irregular C-shaped or stocky branched cells, which are distinct from the curved rod morphology possessed by Δ*csd1* and Δ*csd2 * cells (Bonis *et al.*, 2010 ▶; Sycuro *et al.*, 2010 ▶). Inactivation of Csd3 by an H259A mutation, which is predicted to affect metal coordination in the active site, also resulted in the same degree of morphological abnormality as in Δ*csd3* (Bonis *et al.*, 2010 ▶). *H. pylori* contains high levels of non-cross-linked pentapeptide in the peptidoglycan sacculus (Costa *et al.*, 1999 ▶). The bifunctional peptidase activities of Csd3, together with this observation, makes Csd3 an important regulator of *H. pylori* morphology.

Despite the important roles played by the helical cell shape-determining proteins of *H. pylori* in facilitating stomach colonization, structural reports on them have been very limited. We have recently determined the structure of *H. pylori* Csd4, a Zn^2+^-dependent d,l-CPase and a unique member of the M14 metallopeptidase family (Kim *et al.*, 2014 ▶). It cleaves the bond between γ-d-Glu^2^ and *m*DAP^3^ of the non-cross-linked muramyl tripeptide (muramyl-l-Ala^1^-γ-d-Glu^2^-*m*DAP^3^) of the peptidoglycan to produce the muramyl dipeptide (muramyl-l-Ala^1^-γ-d-Glu^2^) and *m*DAP. Although both Csd3 and Csd4 play essential roles in determining the helical cell shape, their amino-acid sequences are unrelated. Genetic interaction studies between the *csd3* and *csd4* genes revealed that Csd4 d,l-CPase activity does not depend on Csd3 CPase/EPase activity and *vice versa* (Sycuro *et al.*, 2012 ▶).

To provide a structural framework for a better understanding of the molecular function of *H. pylori* Csd3, we report here the crystal structure of N-terminally truncated Csd3 encompassing residues 42–403 (Csd3_Δ41_). It consists of three domains: domain 1 (residues Glu42–Ile124), domain 2 (residues Ile125–Gly228 and Ala360–Phe403) and the C-terminal LytM domain (residues Phe229–Thr359). Csd3 domain 1 and the core of domain 2 (residues Ile125–Gly228) share a common fold despite a very low level of sequence identity. The LytM domain of Csd3 is structurally similar to the corresponding domains of other MEROPS M23 family metallopeptidases. Substrate binding to the active site of the LytM domain is blocked by domain 1 in our structure, suggesting that domain 1 is the inhibitory domain and that our Csd3 structure is in the latent state. The core of domain 2 is held stably against the LytM domain by the C-terminal extended tail region that protrudes from the LytM domain. This work could serve as a foundation for the discovery of novel inhibitors that could prove to be helpful in fighting infections by the major human pathogen *H. pylori*.

## 2. Materials and methods   

### 2.1. Protein expression and purification   

The gene encoding the N-terminally truncated form (residues 42–403; Csd3_Δ41_) of *H. pylori* Csd3 (HP0506 from strain 26695) was PCR-amplified and cloned into the expression vector pET-21a(+) (Novagen) using the NdeI and XhoI restriction-enzyme sites. The recombinant protein, which was fused to a hexahistidine-containing tag (LEHHHHHH) at its C-terminus, was overexpressed in *Escherichia coli* Rosetta 2(DE3)pLysS cells. The cells were grown at 37°C in Terrific Broth culture medium containing 50 µg ml^−1^ ampicillin. Protein expression was induced by 0.5 m*M* isopropyl β-d-1-thiogalactopyranoside and the cells were incubated for an additional 15 h at 30°C. The cells were harvested by centrifugation at 5600*g* for 15 min at 4°C and subsequently lysed by sonication in ice-cold buffer *A* [20 m*M* Tris–HCl pH 7.9, 500 m*M* sodium chloride, 50 m*M* imidazole, 10%(*v*/*v*) glycerol] which was supplemented with 1 m*M* phenylmethylsulfonyl fluoride, 60 m*M* ammonium chloride and 15 m*M* magnesium acetate. The lysate was centrifuged at 36 000*g* for 1 h at 4°C to discard the cell debris. The supernatant was applied onto a HiTrap Chelating HP affinity-chromatography column (GE Healthcare) which was previously equilibrated with buffer *A*. The column was eluted with a linear gradient of imidazole from 50 m*M* in buffer *A* to 500 m*M* in buffer *B*, which consists of 20 m*M* Tris–HCl pH 7.9, 500 m*M* NaCl, 500 m*M* imidazole, 10%(*v*/*v*) glycerol. The Csd3 protein eluted at 125−150 m*M* imidazole concentration was further purified by gel filtration on a HiLoad 16/60 Superdex 200 prep-grade column (GE Healthcare) at two different salt conditions, either with buffer *C* (20 m*M* Tris–HCl pH 7.9, 400 m*M* sodium chloride) or buffer *D* (20 m*M* Tris–HCl pH 7.9, 200 m*M* sodium chloride, 0.1 m*M* zinc chloride). Two different batches of Csd3 yielded different crystal forms, as described below. The purified protein was homogeneous as analyzed by SDS–PAGE. Fractions containing recombinant Csd3 were pooled and concentrated to 10 mg ml^−1^ (0.24 m*M*) for crystallization using an Amicon Ultra-15 Centrifugal Filter Unit (Millipore).

### 2.2. Crystallization   

Crystals were grown at 23°C by the sitting-drop vapour-diffusion method using a Mosquito robotic system (TTP Labtech). We obtained two types of native crystals (form 1 and form 2) under different crystallization conditions. For form 1 crystals, the sitting drops were prepared by mixing 0.4 µl reservoir solution [160 m*M* ammonium sulfate, 80 m*M* sodium acetate pH 4.6, 20%(*v*/*v*) PEG 4000, 20%(*v*/*v*) glycerol] and 0.4 µl purified protein in buffer *C*. Elongated rectangular crystals grew to approximate dimensions of 0.3 × 0.3 × 0.3 mm within a few days. For form 2 crystals, the recombinant Csd3_Δ41_ protein purified in buffer *D* was pre-incubated with buffer *D* supplemented with 1 m*M* zinc chloride under ice for 30 min prior to crystallization setup. Sitting drops were prepared by mixing 0.3 µl reservoir solution [200 m*M* diammonium hydrogen phosphate pH 7.9, 20%(*v*/*v*) PEG 3350] and 0.3 µl protein solution. Hexagonal bipyramidal crystals grew to approximate dimensions of 0.2 × 0.2 × 0.3 mm within a few days.

### 2.3. X-ray data collection and structure determination   

Native X-ray diffraction data were collected on beamline BL-5A at Photon Factory, Japan using an ADSC Q315 CCD detector and on beamline BL-44XU of SPring-8, Japan using a DIP6040 imaging-plate/CCD hybrid detector (Table 1 ▶). Form 1 crystals were flash-cooled in a nitrogen-gas stream at 100 K. They diffracted to 2.0 Å resolution and belonged to space group *P*2_1_2_1_2_1_, with unit-cell parameters *a* = 62.6, *b* = 112.1, *c* = 112.9 Å. Assuming the presence of two Csd3 monomers in the asymmetric unit, the Matthews coefficient and solvent content are 2.33 Å^3^ Da^−1^ and 47.2%, respectively. Form 2 crystals were soaked for several seconds in a cryoprotectant solution consisting of the reservoir solution supplemented with 25%(*v*/*v*) glycerol and were flash-cooled in a nitrogen-gas stream at 100 K. Form 2 crystals diffracted to 1.98 Å resolution and belonged to space group *P*6_5_22, with unit-cell parameters *a* = *b* = 91.5, *c* = 187.0 Å. Assuming the presence of one Csd3 monomer in the asymmetric unit, the Matthews coefficient and solvent content are 2.65 Å^3^ Da^−1^ and 53.6%, respectively.

Selenomethionine-substituted Csd3_Δ41_ protein was not expressed in *E. coli*. Therefore, we prepared a Pt derivative of form 2 crystals by soaking them for 3 min in 2 µl of a heavy-atom-containing cryoprotectant solution, which consisted of the reservoir solution supplemented with 25%(*v*/*v*) glycerol and 30 m*M* K_2_PtCl_4_. Single-wavelength anomalous diffraction (SAD) data were collected at 100 K from the Pt-derivative crystal at a wavelength of 1.0720 Å using an ADSC Q270 CCD detector at the BL-7A experimental station of Pohang Light Source, Republic of Korea. The raw data were processed and scaled using the *HKL*-2000 program suite (Otwinowski & Minor, 1997 ▶). SAD phases were calculated using* AutoSol* from the *PHENIX* software package (Adams *et al.*, 2010 ▶) and were further improved by *RESOLVE* (Terwilliger, 2003 ▶), yielding an interpretable electron-density map at 2.95 Å resolution. Phasing statistics are presented in Table 1 ▶.

### 2.4. Model building and refinement   

*RESOLVE* was also used for autobuilding, which resulted in an initial model accounting for ∼38% of the residues in the recombinant polypeptide chain with much of the sequence assigned. The refined model of the form 2 crystal was used as a search model to determine the Csd3_Δ41_ structure in the form 1 crystal by molecular replacement utilizing *MOLREP* (Vagin & Teplyakov, 2010 ▶). Manual model building was performed using *Coot* (Emsley *et al.*, 2010 ▶) and the models were refined with *REFMAC*5 (Murshudov *et al.*, 2011 ▶), including bulk-solvent correction. A total of 5% of the data was randomly set aside as test data for the calculation of *R*_free_ (Brünger, 1992 ▶). The stereochemistry of the refined models was assessed by *MolProbity* (Chen *et al.*, 2010 ▶). Refinement statistics are presented in Table 1 ▶.

### 2.5. Identification of the bound metal ion by anomalous scattering   

To test whether the metal-binding site is occupied by a Zn^2+^ ion, SAD data were collected from both form 1 and form 2 crystals at 100 K at an X-ray wavelength of 1.2820 Å on beamline 5C of Pohang Light Source (Supplementary Table S1). Anomalous difference maps were calculated using *FFT* (Immirzi, 1966 ▶; Ten Eyck, 1973 ▶) from the *CCP*4 software package.

### 2.6. Equilibrium sedimentation   

Equilibrium sedimentation experiments were performed using a Beckman ProteomeLab XL-A analytical ultracentrifuge in buffer *C* at 4°C. The recombinant Csd3_Δ41_ protein samples were monitored by measuring the absorbance at 280 nm using six-sector cells at three rotor speeds (12 000, 14 000 and 18 000 rev min^−1^, corresponding to 9660, 13 148 and 21 734*g*, respectively, at 6.0 cm radius) and three different protein concentrations (3.77, 5.03 and 6.29 µ*M*). The protein concentration of the recombinant Csd3_Δ41_ was estimated using ∊_280 nm_ = 39 770 *M* ^−1^ cm^−1^ and a molecular mass of 42 562 Da, which includes the C-terminal hexahistidine-containing tag. The partial specific volume of the protein and the buffer density were calculated using *SEDNTERP* (Laue *et al.*, 1992 ▶). Further data manipulation and data analysis by mathematical modelling were performed using *MLAB* (Knott, 1979 ▶).

### 2.7. Accession codes   

The coordinates and structure factors have been deposited in the Protein Data Bank under accession codes 4rny and 4rnz for form 1 and form 2 crystals, respectively.

## 3. Results and discussion   

### 3.1. Structure determination of Csd3   

*H. pylori* Csd3 was predicted to have a single transmembrane helix between Lys7 and Leu26 when its sequence was analyzed by the *TMHMM* server v.2.0 (Krogh *et al.*, 2001 ▶). Therefore, we initially overexpressed the construct comprised of residues 29–403 fused to a C-terminal hexahistidine-containing tag in *E. coli* and the expressed protein was crystallized. However, the crystals diffracted poorly to low resolution (∼4 Å) despite extensive screening of crystallization conditions. This prompted us to try overexpressing a number of shorter constructs encompassing residues 40–403, 40–397, 40–393, 40–390, 42–403, 42–397, 42–393, 42–390, 45–403, 45–397, 45–393 and 45–390. Among these, only the N-terminally 41-residue truncated Csd3 (Csd3_Δ41_) comprised of residues 42–403 was expressed in soluble form, and it yielded two different forms (the orthorhombic form 1 and the hexagonal form 2) of well diffracting crystals (Table 1 ▶).

**Figure 1:**
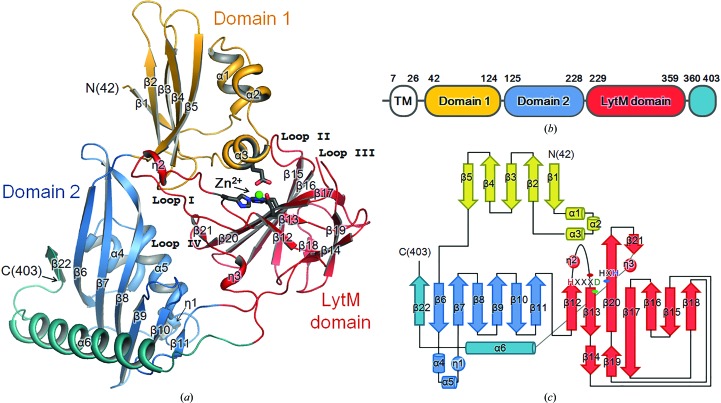
Overall monomer structure and topology of *H. pylori* Csd3_Δ41_. (*a*) Ribbon diagram of the Csd3_Δ41_ monomer (chain *A* of form 1), with the secondary-structure elements labelled. Domain 1, the core of domain 2 and the LytM domain are shown in bright orange, sky blue and red, respectively. The C-­terminal α-helix (α6) and β-strand (β22) are coloured teal. The green sphere is a Zn^2+^ ion. Side chains of the metal-coordinating residues (Glu74, His259, Asp263 and His341) are shown in stick models (dark grey). The secondary structures were defined by *STRIDE* (Heinig & Frishman, 2004 ▶). The walls of the active site in the LytM domain are made up of four loops: loop I (the β12–β13 loop), loop II (the β15–β16 loop), loop III (the β19–β20 loop) and loop IV (the β20–β21 loop). (*b*) Domains of *H. pylori* Csd3 coloured as in (*a*). TM, transmembrane helix. Residue numbers for each domain are indicated. (*c*) Topology diagram of Csd3_Δ41_ coloured as in (*a*). α-Helices, β-strands, 3_10_-helices and loops are shown as cylinders, arrows, circles and solid lines, respectively. Structure figures were drawn using *PyMOL* (DeLano, 2002 ▶).

We have determined the structure of Csd3_Δ41_ (Fig. 1 ▶) using SAD data from a Pt-derivatized form 2 crystal. The model of the form 2 crystal was refined at 1.98 Å resolution to an *R*_work_ and *R*_free_ of 20.8 and 23.9%, respectively (Table 1 ▶). The form 2 crystal contains one monomer of Csd3_Δ41_ in the asymmetric unit. This model of Csd3_Δ41_ excludes six residues (Pro251–Arg256) in a disordered loop near the metal-binding site as well as four residues Gly333–Thr336 and two histidines at the end of the C-terminal affinity tag. The model of the form 1 crystal was refined at 2.00 Å resolution to an *R*_work_ and *R*_free_ of 20.3 and 25.6%, respectively (Table 1 ▶). The form 1 crystal contains two monomers (chains *A* and *B*) of Csd3_Δ41_ in the asymmetric unit. Chains *A* and *B* are related by non­crystallographic twofold symmetry. In both chains *A* and *B*, four residues Gly333–Thr336 and the C-terminal affinity tag (LEHHHHHH) are disordered. Chains *A* and *B* are highly similar to each other, with an r.m.s. deviation of 0.67 Å for 359 C^α^ atoms. However, they show larger structural deviations from the chain of the form 2 crystal, with r.m.s. deviations of 1.61 and 1.85 Å for 353 C^α^ atoms in chains *A* and *B*, respectively. The largest deviations occur in the η1 and α6 helices, with deviations of 6.65 and 5.58 Å at the C^α^ atoms of Pro167 and Gly366, respectively (Supplementary Fig. S1). The observed structural variation is likely to be owing to the inherent flexibility of these regions and also owing to different crystal contacts.

### 3.2. Oligomeric state of Csd3_Δ41_ in solution   

In the form 1 crystal, the two monomers in the asymmetric unit bury a relatively large surface area of 1130 Å^2^ per monomer (6.0% of the monomer surface area), whereas the largest buried surface area in the form 2 crystal is 710 Å^2^ per monomer (3.8% of the monomer surface area). The bulk of crystal interfaces have areas below 1000 Å^2^, with very few representatives above this value (Duarte *et al.*, 2012 ▶). It is also well known that biological interfaces tend to exhibit large areas, with a majority of cases having areas of 1000 Å^2^ and above (Duarte *et al.*, 2012 ▶). This raised a question about the oligomeric state of Csd3_Δ41_ in solution. Therefore, we carried out equilibrium sedimentation experiments. All the measured data fit well to a homogeneous monomer model, indicating that Csd3_Δ41_ exists as monomers in solution at concentrations of up to 6.29 µ*M*. A representative result measured at 18 000 rev min^−1^ using a protein concentration of 3.77 µ*M* is presented in Supplementary Fig. S2.

### 3.3. Three-domain structure of Csd3_Δ41_ and structural similarity searches   

**Figure 2:**
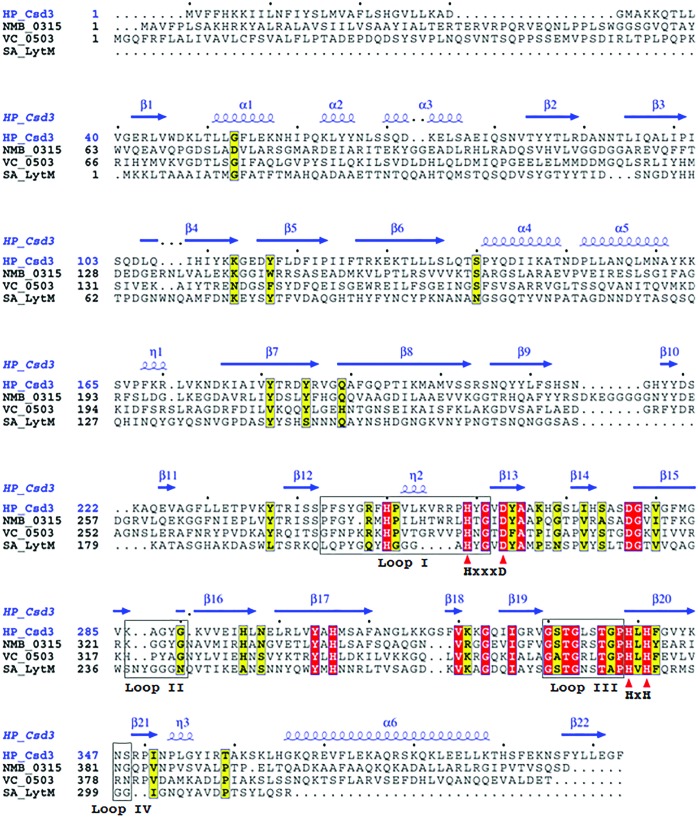
Sequence alignment of four M23B metallopeptidase proteins. Sequence alignment of Csd3 from *H. pylori* strain 26695 (HP0506; HP_Csd3; SWISS-PROT accession code O25247), NMB0315 from *N. meningitidis* (NMB_0315; Q9K163), VC0503 from *Vibrio cholerae* (VC_0503; Q9KUL5) and LytM from *S. aureus* (SA_LytM; O33599). Red triangles indicate the conserved residues in the H*xxx*D and H*x*H motifs (H259-*xxx*-D263 and H339-*x*-H341 in *H. pylori* Csd3) that are important for the metallopeptidase activity. Four loops are indicated by grey boxes.

The structure of Csd3_Δ41_ can be divided into three domains: domain 1 (residues Glu42–Ile124), domain 2 (residues Ile125–Gly228 and Ala360–Phe403) and the LytM domain (residues Phe229–Thr359) (Fig. 1 ▶). A structural similarity search using the *DALI* server (Holm & Rosenström, 2010 ▶) revealed that the entire structure (form 1 crystal, chain *A*) of Csd3_Δ41_ resembles an outer-membrane protein from *Neisseria meningitidis* (NMB0315; PDB entry 3slu; Wang *et al.*, 2011 ▶; r.m.s. deviation of 5.2 Å for 315 equivalent C^α^ positions, *Z*-score of 21.7 and sequence identity of 26%) and a putative lysostaphin peptidase from *Vibrio cholerae* (VC0503; PDB entry 2gu1; Ragumani *et al.*, 2008 ▶; r.m.s. deviation of 4.5 Å for 219 equivalent C^α^ positions, *Z*-score of 18.8 and sequence identity of 23%) (Fig. 2 ▶ and Supplementary Fig. S3). The above two proteins are three-domain proteins, but domain 2 of VC0503 is not included in the structural overlap owing to a large difference in domain arrangements. They belong to the M23B metallopeptidase family (Wang *et al.*, 2011 ▶; Ragumani *et al.*, 2008 ▶).

Domain 1 of Csd3 has an α/β fold consisting of a five-stranded antiparallel β-sheet (β1↓–β2↑–β3↓–β4↑–β5↓) and three short α-helices (Fig. 1 ▶). A cluster of continuously linked helices (α1–α2–α3) is inserted between strands β1 and β2 and is packed on one side of the β-sheet. The core of domain 2 (Ile125–Gly228) also has an α/β fold consisting of a six-stranded antiparallel β-sheet (β6↓–β7↑–β8↓–β9↑–β10↓–β11↑) and three short helices (Fig. 1 ▶). As in domain 1, a cluster of consecutively linked helices (α4–α5–η1) is inserted between strands β6 and β7 and lies on the concave side of the β-sheet. Domain 1 and the core of domain 2 appear to share a common fold despite a very low level of amino-acid sequence identity (Supplementary Fig. S4*a*). However, their charge distributions on the surface are distinct from each other (Supplementary Fig. S4*b*). The C-terminal helix (α6) and β-strand (β22) protruding from the LytM domain are tightly associated with the core of domain 2, covering another face of the β-sheet and extending the β-sheet, respectively.

When a *DALI* search was performed separately against domain 1 (form 1 crystal, chain *A*), the highest structural similarity is observed to the corresponding domains from VC0503 and NMB0315, with *Z*-scores of 7.6 and 5.0, respectively. Besides these, domain 1 displays a very remote structural similarity to single-chain monellin (PDB entry 1mnl; Lee *et al.*, 1999 ▶), with a *Z*-score of 4.2, and human cystatin A (also called stefin A; PDB entry 3kse, chain *D*; M. Renko & D. Turk, unpublished work), with a *Z*-score of 3.5. When the *DALI* search was performed separately against the core of domain 2 (form 1 crystal, chain *A*), the highest structural similarity is observed to the corresponding domains from VC0503 and NMB0315, with *Z*-scores of 14.6 and 14.5, respectively. Besides these, domain 2 exhibits a very remote structural similarity to the core of a single-stranded DNA-binding protein from *Aeropyrum pernix* K1 (PDB entry 4pso; Ghalei *et al.*, 2014 ▶) and perfringolysin O from *Clostridium perfringens* (PDB entry 1m3i; J. Rossjohn, M. Parker, G. Polekhina, S. Feil & R. Tweten, unpublished work), with *Z*-scores of 4.5 and 4.4, respectively.

The bulk of the Csd3 LytM domain is folded as a two-layered sandwich consisting of a larger, seven-stranded antiparallel β-sheet (β12↑–β13↓–β20↑–β17↓–β16↑–β15↓–β18↑) and a smaller, three-stranded antiparallel β-sheet (β14↓–β19↑–β17↓). These β-sheets share a long strand (β17), which is bent like the letter J. Another long strand (β20) associates with a short strand (β21) to form a mini, antiparallel β-sheet (β20↑–β21↓), which is followed by a 3_10_-helix (η3) (Fig. 1 ▶). As expected, the LytM domain (form 1 crystal, chain *A*) of Csd3_Δ41_ exhibits significant structural similarity to the corresponding domains of the M23B metallopeptidases, such as NMB0315 (PDB entry 3slu; r.m.s. deviation of 2.8 Å for 123 equivalent C^α^ positions, *Z*-score of 19.7 and sequence identity of 44%) and VC0503 (PDB entry 2gu1; r.m.s. deviation of 2.7 Å for 120 equivalent C^α^ positions, *Z*-score of 19.0 and sequence identity of 35%). It is also structurally similar to the LytM domains of *Staphylococcus aureus* glycylglycine endopeptidase, another M23B metallopeptidase (PDB entry 2b13; Firczuk *et al.*, 2005 ▶; r.m.s. deviation of 1.9 Å for 118 equivalent C^α^ positions, *Z*-score of 18.3 and sequence identity of 30%), and the virulence factor LasA from *Pseudomonas aeruginosa*, an M23A metallopeptidase (PDB entry 3it7; Spencer *et al.*, 2010 ▶; r.m.s. deviation of 2.1 Å for 107 equivalent C^α^ positions, *Z*-score of 11.4 and sequence identity of 21%). M23A metallopeptidases are distinguished from the more numerous M23B enzymes by structural features that include disulfide bridges and the possession of an additional C-terminal subdomain as well as alterations to the active-site region that are manifested in differences in the sequence spacing between the His and Asp residues of the H*xxx*D motif of the M23B family (Spencer *et al.*, 2010 ▶). In M23A metallopeptidases the intervening sequence is variable and several times longer than that of M23B.

**Figure 3:**
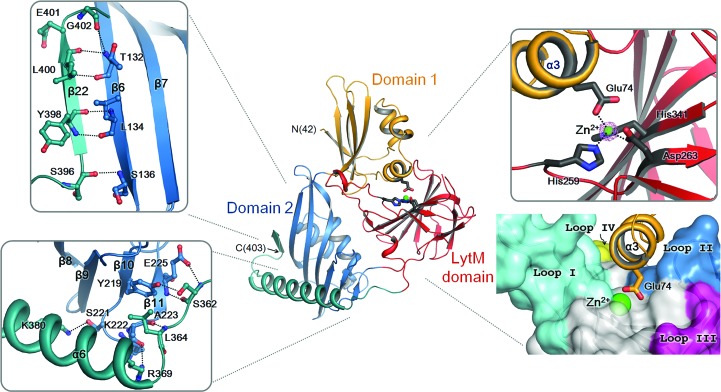
Metal coordination in Csd3_Δ41_ and interactions of the C-terminal α-helix (α6) and β-strand (β22) with the core of domain 2. A ribbon diagram of the Csd3_Δ41_ monomer, coloured as in Fig. 1 ▶(*a*), is shown in the centre. The close-up views on the left represent interactions of the C-terminal strand (β22) with the β6 strand in the core of domain 2 (top) and of the C-terminal helix (α6) with the β-sheet in the core of domain 2 (bottom). Hydrogen-bond interactions are shown as black dotted lines. The close-up views on the right represent the ribbon diagram of the Zn^2+^-binding motif (top) and the surface representation of the substrate-binding groove formed by four loops of the LytM domain (bottom). The electron density for the Zn^2+^ ion in the OMIT *mF*_o_ − *DF*_c_ map is shown as a light pink mesh (contoured at 10σ). To show the detailed interactions more clearly, the close-up views have slightly different orientations.

After leaving the LytM domain, the polypeptide chain is connected to a C-terminal α-helix (α6) and a short β-strand (β22) that fold back onto the core of domain 2, making a tight interaction with the core of domain 2 (Fig. 3 ▶). The α6 helix sits on the opposite side of the β-sheet of domain 2 from the cluster of three helices (α4–α5–η1) and is tightly anchored to the β-sheet through hydrophobic and hydrogen-bond interactions (Fig. 3 ▶). The β22 strand lies antiparallel to the β6 strand of domain 2 and forms the seventh strand of the β-sheet in the core of domain 2 (Fig. 3 ▶). The C-terminal α6–β22 region may play an important role in stabilizing the interdomain orientation of the core of domain 2 and the LytM domain of Csd3.

### 3.4. The active-site structure of the LytM domain   

The Csd3 LytM domain possesses characteristic features of the MEROPS M23 family of metallopeptidases. In these enzymes, the catalytic residues are anchored by the larger antiparallel β-sheet and are grouped around a metal ion (Figs. 3 ▶, 4 ▶ and 5 ▶). We confirmed that this metal-binding site is indeed occupied by a Zn^2+^ ion by calculating anomalous difference maps using anomalous data collected at the zinc absorption edge of 1.2820 Å (Supplementary Table S1 and Supplementary Fig. S5). In all Csd3_Δ41_ structures we observed a tetracoordinated Zn^2+^ ion in the active site, with the expected three amino-acid ligands (His259^∊2^, Asp263^δ1^ and His341^δ1^) and one amino-acid ligand (Glu74^∊2^) from the α3 helix of domain 1 (Fig. 3 ▶). His259 and Asp263 belong to the characteristic **H** *xxx* **D** motif, while His341 is the second histidine of the H*x* **H** motif. The metal–ligand atom distances are in the range 1.95–2.11 Å, consistent with typical Zn^2+^ ion–ligand atom distances. The Zn^2+^-coordinating groups are stabilized by making hydrogen bonds to neighbouring amino-acid residues or water molecules. His259^δ1^ and His341^∊2^ are hydrogen-bonded to the main-chain O atom of Pro243 and a water molecule, respectively. Glu74^∊1^ is hydrogen-bonded to His306^∊2^. His306 of Csd3 is conserved among M23B peptidase proteins (Fig. 2 ▶) and corresponding residues similarly interact with the fourth ligand of the metal ion. For example, His343 in NMB0315 makes a hydrogen bond to a water molecule (Fig. 5 ▶), while His260 in the active LytM from *S. aureus* makes a hydrogen bond to a phosphate ion. If Csd3 were in the active state for peptidase activity, two water molecules should occupy the positions close to the side-chain O atoms of Glu74, as pentacoordination is believed to be consistent with the proposals for the catalytic mechanism (Sabala *et al.*, 2014 ▶).

In Csd3, the distances between the Zn^2+^ and two O atoms from the side chain of Glu74 (Glu74^∊1^ and Glu74^∊2^) are 2.9 and 2.0 Å, respectively. This coordination differs from that in the uncomplexed structure of LasA from *P. aeruginosa* (Spencer *et al.*, 2010 ▶). The Zn^2+^ ion in the uncomplexed structure of LasA is described as pentacoordinated (with a slightly distorted trigonal bipyramidal geometry) with three conserved metal ligands and two conserved water molecules. The distances between Zn^2+^ and the water O atoms (Wat-1 and Wat-2) are 2.1 and 2.7 Å, respectively. In the tartrate-complexed structure of LasA, tartrate O atoms occupy nearly identical positions as both Zn^2+^-coordinated water molecules, with the distances between Zn^2+^ and tartrate O atoms being 2.5 and 2.1 Å, respectively. However, the Zn^2+^ coordination in the tartrate-complexed structure has been described as a tetrahedral geometry (Spencer *et al.*, 2010 ▶). It has been suggested that pentacoordination is consistent with many of the proposals for the catalytic mechanism and that the observations of variable Zn^2+^ coordination among the characterized M23 family metallopeptidases may reflect low energy barriers for changes in Zn^2+^ coordination for catalysis (Sabala *et al.*, 2014 ▶; Spencer *et al.*, 2010 ▶).

**Figure 4:**
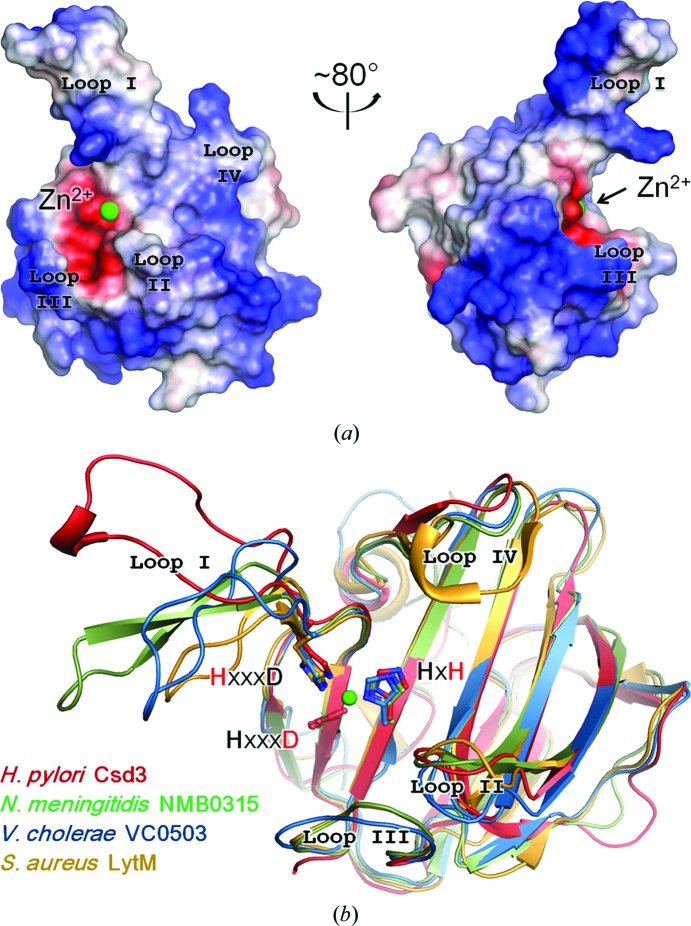
LytM domain of Csd3. (*a*) Two different views of the electrostatic potential surface of the LytM domain of Csd3. The positive and negative electrostatic potentials on the surface are coloured blue and red, respectively. Four loops that form the substrate-binding groove around the Zn^2+^ ion (green sphere) are denoted by loops I–IV. (*b*) Superposition of LytM domains in four M23B metallopeptidases. The LytM domains of *H. pylori* Csd3 (red), *N. meningitidis* NMB0315 (pale green; PDB entry 3slu), *V. cholerae* VC0503 (sky blue; PDB entry 2gu1) and *S. aureus* LytM (yellow/orange; PDB entry 1qwy) are superimposed and shown as ribbon diagrams.

**Figure 5:**
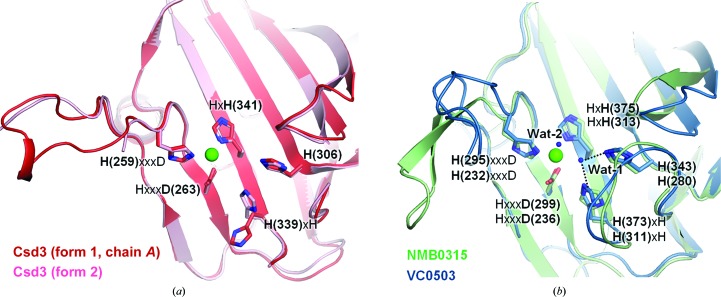
Conserved residues in the active site of M23B metallopeptidases. (*a*) Superposition of LytM domains in two crystal forms of *H. pylori* Csd3. Chain *A* of the form 1 crystal (red) and the form 2 crystal (pink) are shown as ribbon diagrams. (*b*) Superposition of the LytM domains in two M23B family members (NMB0315 and VC0503) coloured as in Fig. 4 ▶(*b*). Conserved residues are shown as stick models and are labelled (NMB0315 at the top and VC0503 at the bottom). Metal ions and water molecules are shown as green spheres and blue dots, respectively. In NMB0315, the Zn^2+^ ion was replaced by an Ni^2+^ ion during affinity chromatography (Wang *et al.*, 2011 ▶). Wat-1 and Wat-2 are present in NMB0315, where the Ni^2+^ ion is pentacoordinated. Black dotted lines denote hydrogen bonds to Wat-1 in NMB0315. In VC0503, the Zn^2+^ ion is tetracoordinated, with one water molecule (omitted for clarity) located between Wat-1 and Wat-2.

The floor of the substrate-binding groove of the LytM domain is built by the larger β-sheet and the walls of the active site are made up of four loops: loop I (the β12–β13 loop), loop II (the β15–β16 loop), loop III (the β19–β20 loop) and loop IV (the β20–β21 loop) (Figs. 2 ▶, 3 ▶ and 4 ▶). A superimposition of the LytM domain of Csd3 with those of NMB0315, VC0503 and *S. aureus* LytM reveals that the larger β-sheets deviate little from each other, but that the difference is larger for loops I–IV (Fig. 4 ▶ *b*). Loop I of Csd3 shows the most significant deviation among the homologues. In form 1 crystals, it is involved in crystal-packing interactions by forming sulfate-mediated salt bridges (Supplementary Fig. S6). In form 2 crystals, it is not involved in crystal packing and is disordered. Therefore, we conclude that the observed structural difference of loop I is largely owing to differences in crystal packing and also to its inherent flexibility. In all structures of Csd3_Δ41_, three residues (Gly333-Leu334-Ser335) of loop III are disordered. Loop III has significant sequence conservation and immediately precedes the H*x*H motif (Fig. 2 ▶). Gly333 and Ser335 of Csd3 are conserved, whereas Leu334 in Csd3 is substituted by Arg368 and Arg365 in NMB0315 and VC0503, respectively, and Asn286 in *S. aureus* LytM (Fig. 2 ▶). In the structure of the active LytM from *S. aureus* (PDB entry 2b44), a phosphate ion, as a substrate candidate, is fixed in space in the active site by multiple hydrogen bonds to Asn286 (Firczuk *et al.*, 2005 ▶). Ser369 of NMB0315 and Ser287 of the active LytM from *S. aureus*, corresponding to the disordered Ser335 in loop III of Csd3, interact with the first histidine of the H*x*H motif. This histidine is considered to be a catalytic residue by coordinating a Zn^2+^-bound water molecule that acts as the nucleophile in the hydrolytic reaction (Spencer *et al.*, 2010 ▶). Interestingly, the side chain of His339 of Csd3, the first histidine of the H*x*H motif, shows two different orientations. Its orientation in the form 2 crystals is similar to those in other homologous proteins (Fig. 5 ▶). However, it is flipped about 104° towards the solvent in the form 1 crystals (Fig. 5 ▶).

### 3.5. Domain 1 occludes the active site of the LytM domain   

**Figure 6:**
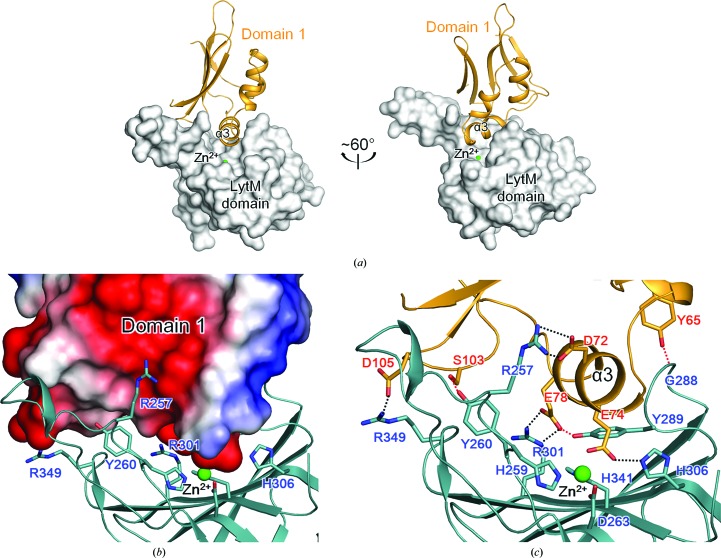
The active site of the Csd3 LytM domain is blocked by domain 1. In this figure, domain 2 is omitted for clarity. (*a*) Two different views of the interaction between domain 1 (shown as bright orange ribbons) and the LytM domain (shown as a grey surface diagram) of Csd3. (*b*) Electrostatic surface diagram of domain 1 and a ribbon diagram of the LytM domain (coloured deep teal). Residues of the LytM domain located at the domain interface are shown as stick models and are labelled. (*c*) Ribbon diagram of domain 1 (bright orange) and the LytM domain (coloured deep teal). Residues at the domain interface are shown as stick models. Hydrogen-bond interactions and salt-bridge interactions are shown as red and black dotted lines, respectively.

In both the form 1 and the form 2 crystals, domain 1 blocks the active-site cleft of the LytM domain (Fig. 6 ▶), with the protruding helix α3 contributing to the Zn^2+^ coordination sphere. Several negatively charged residues from domain 1 (Asp72, Glu74, Glu78 and Asp105) and positively charged residues (Arg257, Arg301 and Arg349) as well as the Zn^2+^ ion from the LytM domain form strong salt bridges at the interface between these domains. As described above, the Zn^2+^ ion in the active site of the LytM domain is tetrahedrally coordinated by the three conserved residues (His259 and Asp263 of the H*xxx*D motif and the second histidine His341 of the H*x*H motif) of the LytM domain as well as the nonconserved Glu74 in helix α3 of domain 1. There is a hydrogen bond between Glu74 and the conserved His306 of the LytM domain. Asp72 interacts with Arg257 in loop I of the LytM domain by forming a salt bridge. Glu78 forms a salt bridge with Arg301 in the β17 strand of the LytM domain; it also makes a hydrogen bond to Tyr289 in loop II of the LytM domain. In addition, Asp105 in the β3–β4 loop of domain 1 forms a salt bridge with Arg349 in the β21 strand of the LytM domain. Tyr65 in the α2 helix of domain 1 forms a hydrogen bond to the main-chain N atom of Gly288 in loop II of the LytM domain. Ser103 in the β3–β4 loop of domain 1 is hydrogen-bonded to Tyr260 in the β13 strand of the LytM domain. On the basis of these extensive interactions between domain 1 and the LytM domain, we suggest that Csd3_Δ41_ is in an auto-inhibited state in the crystal. This is similar to the occlusion of the active site by an N-terminal segment in structurally related three-domain proteins in the inhibited conformational state (Supplementary Fig. S7). In NMB0315, the N-terminal short β3–β4 loop stretches into the active site and tightly associates with the catalytic domain (Wang *et al.*, 2011 ▶). In VC0503, an N-terminal helix (α2) occupies the active-site cleft (Ragumani *et al.*, 2008 ▶). In both cases the loop or helix does not participate in metal coordination.

The Zn^2+^ ion bound to the active site of Csd3 is tetrahedrally coordinated by the side chains of four residues (Glu74, His259, Asp263 and His341) without any ordered water molecules. Glu74 is not conserved, whereas His259, Asp263 and His341 are conserved (Fig. 2 ▶). The ligand position corresponding to the nonconserved Glu74 is usually occupied by a catalytic water molecule or various anions in the active forms of other M23 metallopeptidases (Supplementary Fig. S7). In the case of LytM from *S. aureus*, a truncated version that lacks Asn117 has a much higher specific activity than the full-length enzyme, in which the poorly conserved Asn117 of the inhibitory domain occupies one of the ligand sites of Zn^2+^ (Odintsov *et al.*, 2004 ▶). In the active, truncated LytM from *S. aureus*, Asn117 is replaced by phosphate, cacodylate or tartrate from the crystallization buffer (Firczuk *et al.*, 2005 ▶)*.*

## 4. Discussion   

We have determined the first crystal structure of Csd3 from *H. pylori*, a protein that influences the helical cell shape crucial for the survival of *H. pylori* in the stomach. We note that the fold of Csd3 domain 1 is very remotely related to monellin/cystatin superfamily proteins, some of which act as inhibitors of cysteine peptidases. The active site of the C-terminal LytM domain is blocked by the inhibitory domain 1, in particular helix α3 with the Zn^2+^-coordinating Glu74. The structure of an AmiB orthologue from *Bartonella henselae* revealed that the active site of AmiB is similarly occluded by a conserved α-helix with a Zn^2+^-coordinating glutamate residue (Glu290) and it was suggested that auto-inhibition is a critical feature of the regulation of peptidoglycan amidases required for cell division in Gram-negative bacteria (Yang *et al.*, 2012 ▶). We measured the CPase activity of *H. pylori* Csd3 against the synthetic muramyl pentapeptide (the substrate for the CPase activity of Csd3) using mass analysis. The muramyl pentapeptide (5 m*M*) was incubated at 37°C for 150 min with recombinant Csd3_Δ41_ (5 µ*M*) in the presence of 2.5 m*M* metal ion (either Zn^2+^ or Mg^2+^) or 2.5 m*M* EDTA. We used two different buffers for dissolving the reaction mixture: (i) 20 m*M* HEPES pH 7.9, 100 m*M* NaCl or (ii) 20 m*M* sodium phosphate pH 6.0. No reaction product was observed in all of the reaction conditions that we tested. As a positive control, we could detect the muramyl tetrapeptide product when the muramyl pentapeptide was incubated with *Enterococcus faecium* VanY. This result is consistent with a latent, inactive state of Csd3_Δ41_ in the crystal.

Our work suggests that the inhibitory domain 1, including the α3 helix, should be displaced from the active site of the LytM domain for activation of the latent Csd3. The activation of *H. pylori* Csd3 may occur by autoproteolysis or may require proteolytic cleavage by other endopeptidases to free the catalytic LytM domain from the inhibitory domain 1. When we tried limited proteolysis with trypsin, chymotrypsin and pepsin, no discrete cleavage at specific sites took place. To physically remove the inhibitory domain 1 from Csd3, we tried to overexpress various constructs (residues 80–403, 81–403, 83–403, 124–403, 125–403, 127–403, 130–403, 227–403, 230–403, 233–403, 125–359, 127–359, 227–359, 230–359 and 233–359) that lacked helix α3. However, none of them was expressed in *E. coli* in a soluble form. Alternatively, a large conformational changed induced by an allosteric regulator may lead to opening of the active site. For instance, a large conformational change culminates in opening of the active site to permit substrate entry when an allosteric site ∼60 Å distant from the d,d-transpeptidase active site is occupied in penicillin-binding protein 2a (PBP2a) from *S. aureus* (Otero *et al.*, 2013 ▶).

To provide details of the interaction of the substrate with Csd3, we soaked the form 1 and form 2 crystals in a cryoprotectant solution that was supplemented with the muramyl pentapeptide. No electron density for the substrate peptide was observed. We also tried to co-crystallize Csd3_Δ41_ in the presence of excess muramyl pentapeptide under conditions similar to the crystallization conditions for form 1 and form 2 crystals, but this attempt did not produce any crystals. Owing to a limited supply of the muramyl pentapeptide, it was not possible to perform more extensive screening of the crystallization conditions in its presence. Co-crystallization of Csd3_Δ41_ in the presence of cross-linked dimers of muramyl pentapeptides and tetrapeptides (the substrate for the EPase activity of Csd3) was not possible because this substrate is neither commercially available nor easy to synthesize.

*H. pylori* Csd1 (HP1543), Csd2 (HP1544) and Csd3 (HP0506) contain a LytM domain of the M23B metallopeptidase family; they all catalyze the same reaction as EPases. Csd2 could be a nonpeptidase homologue of the M23B family, as the first histidines of the characteristic H*xxx*D and H*x*H motifs in its LytM domain are mutated to Glu at residue 165 and Lys at residue 246, respectively. Csd3 exhibits CPase activity, while Csd1 may or may not have such an activity. *H. pylori* Csd3 (with 403 residues) is considerably longer in sequence compared with Csd1 (312 residues) and Csd2 (308 residues). Sequence alignment of Csd1 and Csd2 with either domain 1 or the core of domain 2 of Csd3 reveals that the proregions of both Csd1 and Csd2 have higher levels of sequence similarity to domain 1. The significantly shorter lengths of Csd1 and Csd2 indicates the presence of only one domain in their proregions. This may affect their CPase activity. However, no information on the three-dimensional structures of Csd1 and Csd2 is available at present and thus structural studies on them are required to compare the structures of Csd1 and Csd2 with Csd3 and to understand the functional differences in structural terms.

Bacteria may change their morphology to fit the circumstances for survival (Young, 2007 ▶). Under stressed conditions such as subinhibitory concentrations of antibiotics, *H. pylori* is able to enter a viable but nonculturable state, in which the microorganism modifies its morphology from a spiral to a coccoid form (Cellini, 2014 ▶). The viable coccoid form is more persistent in the host and environment (Cellini, 2014 ▶). It has been reported that overproduction of Csd3/HdpA in *H. pylori* strain N6 led to a transformation from rod-shaped to viable cocci-shaped bacteria (Bonis *et al.*, 2010 ▶). This raises the possibility that transition of the helical shape to a coccoid shape may be associated with the overexpression of Csd3. If this is the case, Csd3 could be an attractive drug target not only for eradicating helical-shaped *H. pylori* but also for inhibiting the morphological transformation into the persistent coccoid form.

## Supplementary Material

PDB reference: Csd3, 4rny

PDB reference: 4rnz

Supporting Information.. DOI: 10.1107/S1399004715000152/tz5068sup1.pdf

## Figures and Tables

**Table 1:** Data-collection and refinement statistics Values in parentheses are for the highest resolution shell.

Data set	Form 1	Form 2	Pt (peak)
Data collection			
Beamline and source†	BL-5A, PF	BL-44XU, SPring-8	BL-7A, PLS
Space group	*P*2_1_2_1_2_1_	*P*6_5_22	*P*6_5_22
Unit-cell parameters			
*a* ()	62.6	91.5	92.0
*b* ()	112.1	91.5	92.0
*c* ()	112.9	187.0	186.5
= ()	90	90	90
()	90	120	120
X-ray wavelength ()	1.0000	0.9000	1.0720
Resolution range ()	50.02.00 (2.032.00)	50.01.98 (2.011.98)	30.02.95 (3.002.95)
Total/unique reflections	264154/54371	350996/33042	480902/18708‡
Completeness (%)	99.5 (100.0)	99.9 (100.0)	100.0 (100.0)‡
*I*/(*I*)	27.5 (2.9)	41.9 (3.5)	64.0 (13.0)‡
*R*_merge_ § (%)	8.3 (66.9)	8.8 (87.7)	12.9 (58.2)‡
CC_1/2_ ¶ (%)	99.7 (75.9)	99.9 (91.6)	99.9 (92.6)
SAD phasing			
Figure of merit (before/after density modification)			0.39/0.73
Model refinement			
PDB code	4rny	4rnz	
Resolution range ()	50.02.00	50.01.98	
*R*_work_/*R*_free_ †† (%)	20.3/25.6	20.8/23.9	
No. of non-H atoms			
Protein	5822	2919	
Metal ion‡‡	2	3	
Water oxygen	225	148	
Glycerol	30	18	
Sulfate ion	45		
Phosphate ion		15	
Average *B* factor (^2^)			
Protein	37.0	40.4	
Metal ion‡‡	26.1	33.7	
Water oxygen	34.6	40.8	
Glycerol	49.3	55.0	
Sulfate ion	2.3		
Phosphate ion		65.0	
R.m.s. deviations from ideal geometry			
Bond lengths ()	0.013	0.009	
Bond angles ()	1.58	1.42	
R.m.s. *Z*-scores§§			
Bond lengths	0.64	0.46	
Bond angles	0.73	0.64	
Ramachandran plot¶¶			
Favoured/outliers (%)	96.9/0.0	96.3/0.0	
Poor rotamers (%)	0.16	0.00	

† PF, Photon Factory, Japan; PLS, Pohang Light Source, Republic of Korea.

‡ Friedel pairs were treated as separate observations.

§ *R*_merge_ = 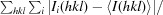 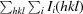, where *I*(*hkl*) is the intensity of reflection *hkl*, 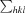 is the sum over all reflections and 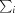 is the sum over *i* measurements of reflection *hkl*.

¶ CC_1/2_ is the correlation coefficient of the mean intensities between two random half-sets of data.

†† *R*_work_ = 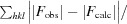 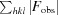, where *R*_free_ is calculated for a randomly chosen 5% of reflections which were not used for structure refinement and *R*_work_ is calculated for the remaining reflections.

‡‡ The two metal ions in form 1 are Zn^2+^ ions in the active site of two chains of Csd3_41_. Form 2 contains a Zn^2+^ ion in the active site and is likely to contain two Ni^2+^ ions bound to the C-terminal hexahistidine tag.

§§ Values obtained using *REFMAC*5.

¶¶ Values obtained using *MolProbity*.
