# Supplementary material for: Structure of Csd3 from Helicobacter pylori, a cell shape-determining metallopeptidase
Source: Acta Crystallogr D Biol Crystallogr. 2015 Feb 26;71(Pt 3):675–86. doi: 10.1107/S1399004715000152 (PMC4356371; doi:10.1107/S1399004715000152)
Supplement: Supplementary file 1 [file d-71-00675-sup1.pdf]

BIOLOGICAL
CRYSTALLOGRAPHY

Volume 71 (2015)

Supporting information for article:

Structure of Csd3 from *Helicobacter pylori*, a cell-shape determining metallopeptidase

Doo Ri An, Hyoun Sook Kim, Jieun Kim, Ha Na Im, Hye Jin Yoon, Ji Young Yoon, Jun Young Jang, Dusan Hesek, Mijoon Lee, Shahriar Mobashery, Soon-Jong Kim, Byung Il Lee and Se Won Suh

**Table S1. Statistics on data collection of Zn SAD data sets**		
Data set	Zn SAD (Form 1)	Zn SAD (Form 2)
Space group	$P2_12_12_1$	$P6_522$
Unit cell lengths, a, b, c (Å)	62.2, 113.0, 112.7	92.4, 92.4, 186.7
Unit cell angles, α, β, γ (°)	90, 90, 90	90, 90, 120
X-ray wavelength (Å)	1.2820	1.2820
Resolution range (Å)	50.0–2.30 (2.34–2.30)[a]	50.0–2.35 (2.39–2.35)[a]
Total / unique reflections	559,489 / 68,296[b]	544,542 / 36,489[b]
Completeness (%)	100.0 (99.6)[a,b]	99.1 (97.4)[a,b]
$<I> / <\sigma_I>$	57.2 (3.6)[a,b]	65.4 (8.7)[a,b]
R_{merge}[c] (%)	8.8 (83.9)[a,b]	14.4 (72.4)[a,b]
$CC_{1/2}$[d] (%)	99.8 (85.3)[a]	99.5 (86.5)[a]

[a] Values in parentheses refer to the highest resolution shell.
[b] Friedel pairs were treated as separate observations.
[c] $R_{merge} = \Sigma_h\Sigma_i \mid I(h)_i - <I(h)> \mid / \Sigma_h\Sigma_i I(h)_i$, where $I(h)$ is the intensity of reflection h, Σ_h is the sum over all reflections, and Σ_i is the sum over i measurements of reflection h.
[d] $CC_{1/2}$ is the correlation coefficient of the mean intensities between two random half-set of data.

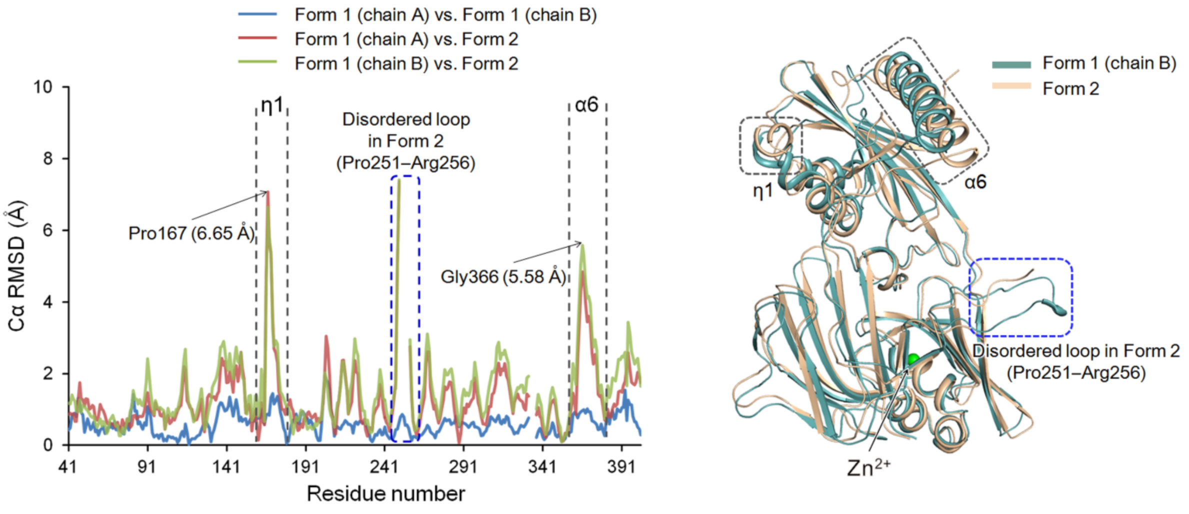

(*a*) (*b*)

Supplementary Figure S1

Comparisons of Csd3$_{\Delta41}$ monomer structures. (*a*) Plot of Cα r.m.s. deviations for pairwise comparisons among three monomer models of Csd3$_{\Delta41}$. Chains A and B of Form 1 crystal are more similar to each other than they are to Form 2 crystal. The η1 and α6 helices show large deviations between Form 1 and Form 2 crystals. (*b*) A superposition of two monomer models of Csd3$_{\Delta41}$. Models of Form 1 crystal (chain B) and Form 2 crystal are colored in teal blue and light orange, respectively. The η1 and α6 helices, which show significant conformational differences, are highlighted by gray dotted boxes. Residues Pro251–Arg256 are disordered in Form 2 crystal and are highlighted by a blue dotted box.

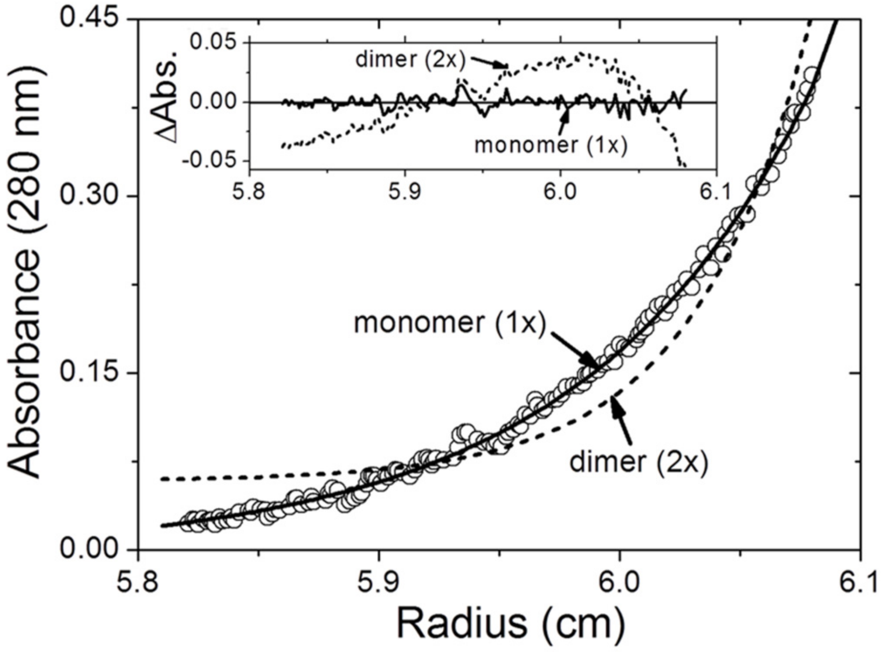

Supplementary Figure S2

Sedimentation equilibrium distributions of *H. pylori* Csd3$_{\Delta 41}$. Representative data measured at 18,000 rpm using the 3.77 μM protein concentration are shown. The circles are experimental absorbance data at 280 nm and the solid line is a fitting line for a homogeneous monomer (1×) model. The dotted line is a fitting line for an ideal homogeneous dimer (2×) model. (Inset) Distributions of the residuals for monomer (1×, solid line) and dimer (2×, dotted line) models, respectively. Random distribution of the residuals for the monomer (1×) model indicates that Csd3$_{\Delta 41}$ exists as homogeneous monomers in solution.

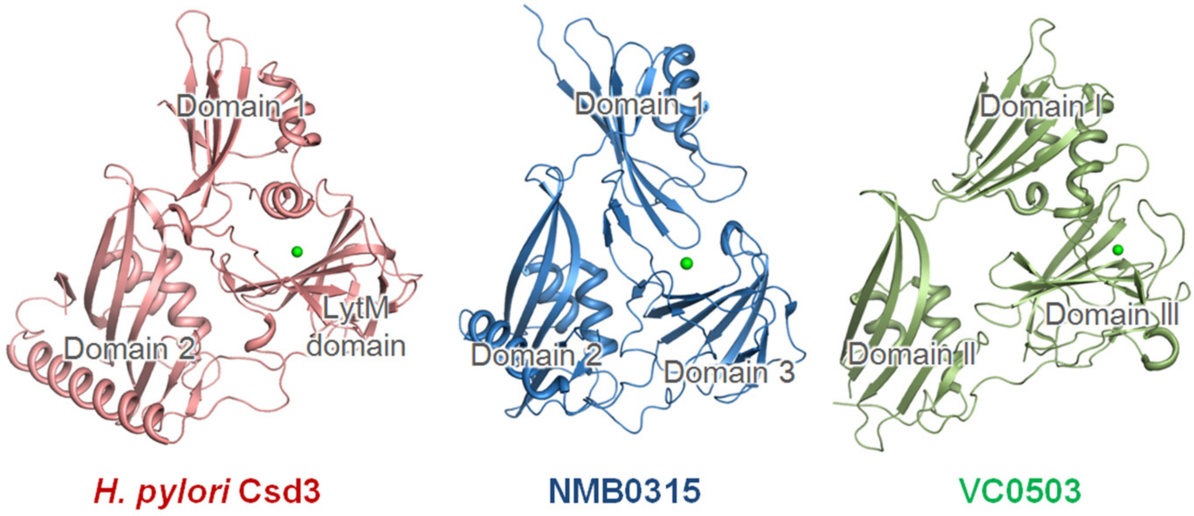

Supplementary Figure S3

Ribbon diagram of three-domain proteins showing structural similarity to the entire structure of *H. pylori* Csd3$_{\Delta 41}$. The structure of *H. pylori* Csd3$_{\Delta 41}$ (deep salmon) is on the left, *N. meningitides* NMB0315 (sky blue; PDB code, 3SLU) in the middle, and *V. cholera* VC0503 (pale green; PDB code, 2GU1) on the right. Metal ions are shown as green spheres. In NMB0315, the Zn^{2+} ion was replaced with a Ni^{2+} ion during affinity chromatography (Wang *et al*., 2011).

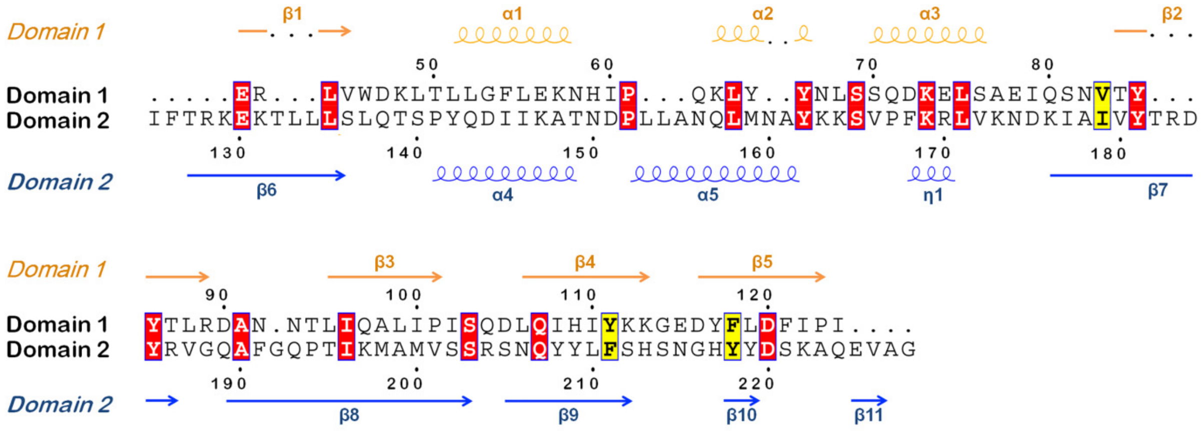

(*a*)

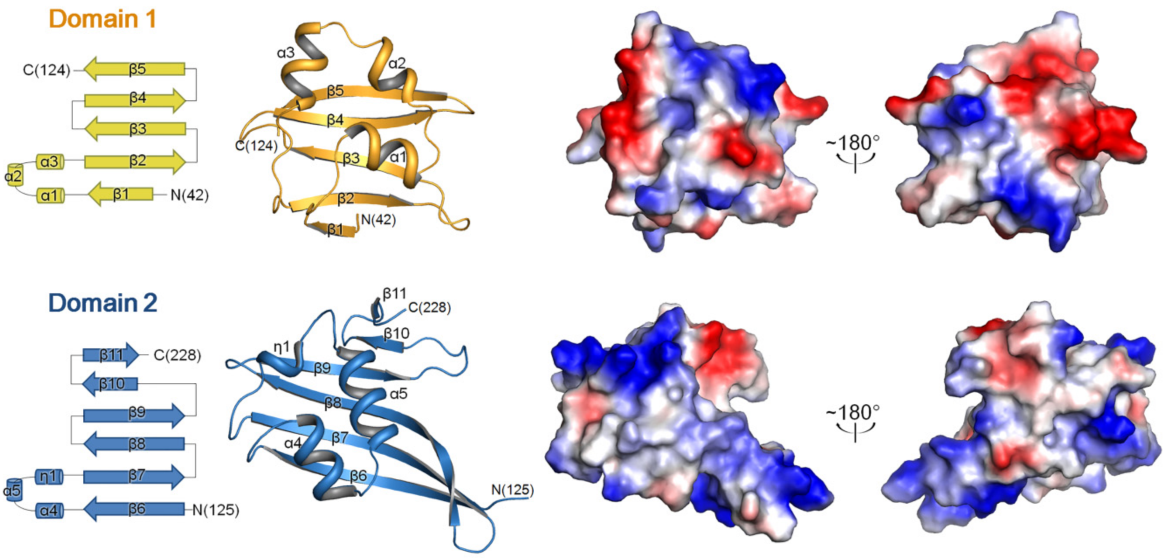

(*b*)

Supplementary Figure S4

Sequence and structure comparison of Csd3 domain 1 and domain 2. (*a*) Sequence alignment of Csd3 domain 1 and domain 2 with secondary structures. (*b*) Comparison of charge distributions on the surface of Csd3 domain 1 (top) and domain 2 (bottom). Topology and ribbon diagrams for domain 1 and domain 2 are colored as in Fig. 1*a*, with the secondary structure elements labeled. Two different views of the electrostatic potential surface diagrams related by a 180° rotation are shown next to the ribbon diagrams.

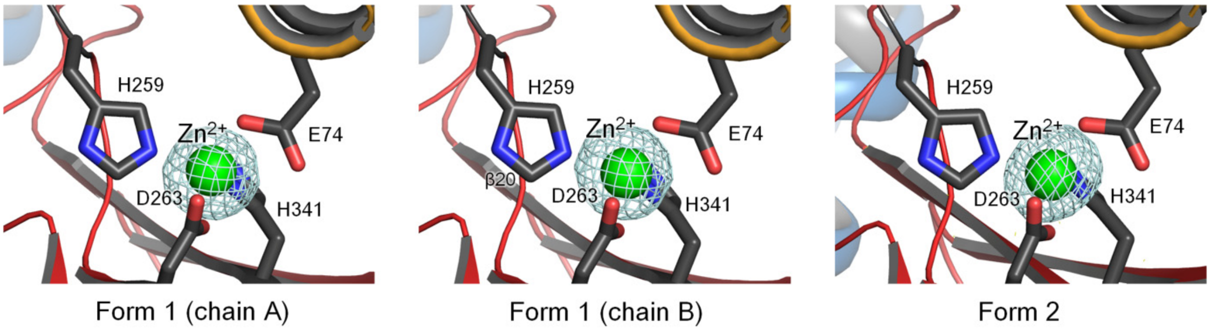

Supplementary Figure S5

Electron density of metal ions bound to the Zn^{2+}-binding site in the anomalous difference Fourier maps (contoured at 10σ and colored in cyan), calculated using the Zn SAD data sets from Form 1 and Form 2 crystals (Supplementary Table S1). Ribbon diagrams are colored as in Fig. 1*a*.

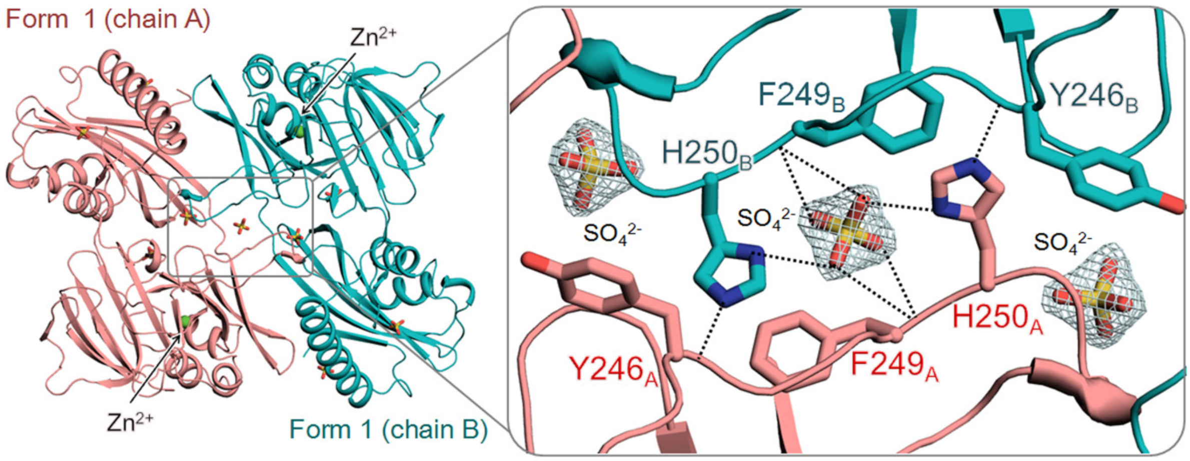

Supplementary Figure S6

Crystal packing interactions in Form 1 crystal of *H. pylori* $Csd3_{\Delta41}$. Chain A (salmon) and chain B (teal blue) of $Csd3_{\Delta41}$ in the asymmetric unit are shown in ribbon diagrams. Zn^{2+} ions are indicated by green spheres. Nine sulfate ions are shown in stick models. A close-up view on the right represents inter-chain interactions mediated by a sulfate ion at the noncrystallographic two-fold symmetry axis. Residues at the interface between two chains are shown in stick models and labeled. The electron density for the sulfate ions in the $2mF_o - DF_c$ map are shown in cyan colored mesh (contoured at 2σ). Hydrogen bonds and salt bridges are indicated by black dotted lines.

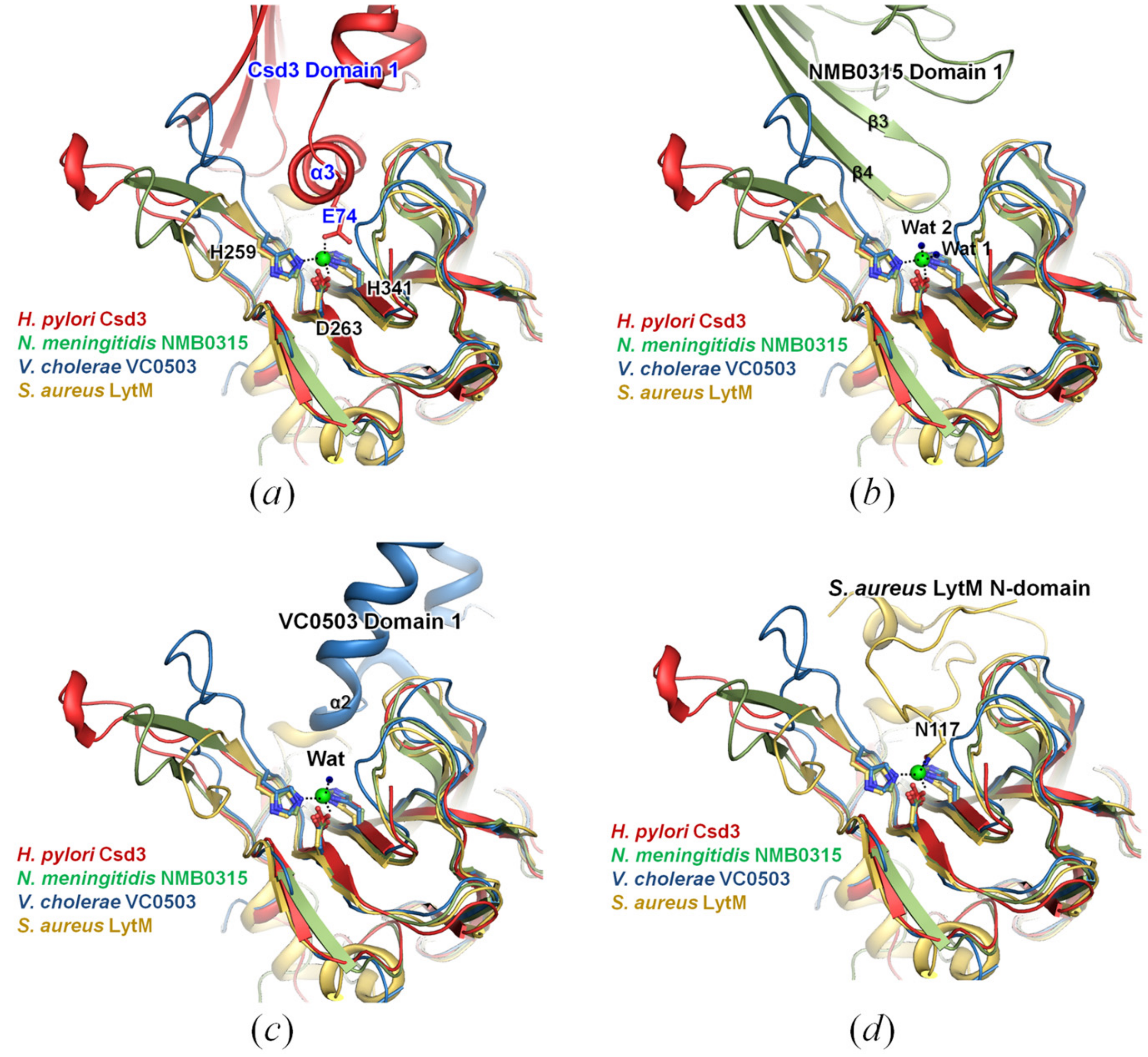

Supplementary Figure S7

Structural superposition of LytM domains of M23B family metallopeptidases in the inhibited state. Four LytM domains are superimposed as in Fig. 4*b* but are shown in a different orientation. (*a-d*) Domain 1 of *H. pylori* Csd3 (*a*), and corresponding inhibitory domains of *N. meningitidis* NMB0315 (*b*), *V. cholerae* VC0503 (*c*), and *S. aureus* LytM (*d*) are shown in addition to the superposed LytM domains. Metal ions are shown as green spheres. In NMB0315, the Zn^{2+} ion was replaced with a Ni^{2+} ion during affinity chromatography (Wang *et al*., 2011). Water molecules are shown as blue dots. Black dotted lines denote metal coordination.

Reference

Wang, X., Yang, X., Yang, C., Wu, Z., Xu, H. & Shen, Y. (2011). *PLoS One* **6**, e26845.
